# Comparison of Ceramic-on-Ceramic vs. Ceramic-on-Polyethylene for Primary Total Hip Arthroplasty: A Meta-Analysis of 15 Randomized Trials

**DOI:** 10.3389/fsurg.2021.751121

**Published:** 2021-12-16

**Authors:** Yan Fang, Xiaobin Shang

**Affiliations:** ^1^Department of Anesthesiology, Tongji Hospital, Tongji Medical College, Huazhong University of Science and Technology, Wuhan, China; ^2^Department of Orthopaedics, Tongji Hospital, Tongji Medical College, Huazhong University of Science and Technology, Wuhan, China

**Keywords:** ceramic on ceramic, ceramic on polyethylene, meta-analysis, total hip, review–systematic

## Abstract

**Objective:** This meta-analysis aimed to compare ceramic-on-ceramic (COC) components and ceramic-on-polyethylene (COP) components during total hip arthroplasty (THA).

**Settings:** A meta-analysis of randomized controlled trials (RCTs) comparing COC and COP during primary THA was conducted. Electronic searches were current to March 2021.

**Participants:** Trials were included for meta-analysis if they compared at least the bearing surfaces of COC and COP for patients undergoing primary THA and if they reported the outcomes of THA after a certain period of follow-up and only RCTs in English were included.

**Primary and Secondary Outcome Measures:** The primary endpoints consist of audible noise, prosthesis fracture, and revision. Secondary endpoints include dislocation, deep infection, osteolysis, and prosthesis loosening. Extracted data were statistically analyzed with the Stata11.0.

**Results:** A total of 15 RCTs containing 2,702 patients (2,813 hips) were included in this study. The audible noise [odds ratio (OR) = 5.919; 95% CI: 2.043, 17.146; *p* ≤ 0.001] and prosthesis fracture (OR = 35.768; 95% CI: 8.957, 142.836; *p* = 0.001) were significantly higher in the COC group. Hip function, revision rate, dislocation rate, deep infection rate, osteolysis rate, and prosthesis loosening rate were comparable between these two groups, while the wear rate was higher in the COP group.

**Conclusion:** This study indicated comparable outcomes of COC and COP bearing surfaces in primary THA; high-quality RCTs with a long-term follow-up are still urgently needed to provide more evidence on the optimal bearing surfaces in primary THA.

## Introduction

Total hip arthroplasty (THA) is currently known as one of the most mature surgical procedures to treat diverse hip diseases including end-stage inflammatory or degenerative arthritis, displaced fractured neck of the femur, and end-stage avascular necrosis (1, 2). Up to date, more than 1 million THAs have been performed every year worldwide and the number in the United States alone was more than 5,00,000 (3). Despite the tremendous advances in prosthetic design and materials over the past decades, heated debates with respect to the choice of bearing surfaces remain controversial, especially about ceramic-on-ceramic (COC) components and ceramic-on-polyethylene (COP) components, both of which are most commonly used in clinical work (4). Since ceramic material was first introduced in THA by Boutin 50 years ago, it has attracted the attention of people owing to the typical feature of hardness and wettability with high resistance against wear (5). As the typical representative of “hard-on-hard” bearing surfaces, COC was successful in resolving the problem of wear and wear-related problems such as osteolysis (6), prosthetic loosening (7), and prosthetic joint infection (PJI) (8). As a result, the COC bearing surface is an optimal choice for younger patients with more activity. Compared with COC, COP was reported with higher wear rates and this may be the main concern in young patients and can be a choice for old patients and young patients with a possible contraindication of COC such as severe developmental dysplasia of the hip and posttraumatic acetabular deformity (9). However, patients often complained about COC due to obvious noise from clashing ceramics, which rarely occurs in COP (10). More importantly, given ceramic is more fragile than polyethylene (PE) in essence, both the patients and surgeries may concern fractures of the ceramic lining (11). Therefore, it is still hard to decide the optimal manner considering the complex factors such as the age of the patient (12), specific etiology (13), surgical approach (14), and follow-up time (15), all of which may affect the final result and interfere with the effect appraisal of prosthetic bearing surfaces. Therefore, the purpose of the present meta-analysis was to compare the outcomes of these two bearing surfaces in THA according to the selected randomized controlled trials (RCTs) and this can provide meaningful clinical guidance for the selection of prostheses.

## Materials and Methods

This meta-analysis was performed based on the Preferred Reporting Items for Systematic Reviews and Meta-Analyses (PRISMA) guidelines (16).

### Patient and Public Involvement

No patient was involved.

### Literature Search

We searched all the RCTs that compared COC vs. COP for THA through electronic databases including PubMed, Google Scholar, Embase, and the Cochrane library with a full text in English. The retrieval was current to March 2021. The following keywords “ceramic-on-ceramic or COC,” “ceramic-on-polyethylene or COP,” and “total hip arthroplasty or total hip replacement” with the Boolean operators were set as the search strategy. Furthermore, bibliographies of studies were manually screened for potential eligible trials. Additional attention was given to the references from the meta-analyses comparing bearing surfaces in THA.

### Inclusion and Exclusion Criteria

The inclusion criteria of this meta-analysis were as follows: (1) comparing at least the bearing surfaces of COC and COP for patients undergoing primary THA; (2) reporting the outcomes of THA after a certain period of follow-up; (3) published in English; and (4) only RCTs were included.

We excluded studies that were of low quality or did not follow inclusion criteria. To obtain comprehensive literature, prosthesis fixation, prosthesis manufacturer, prosthesis size, follow-up time, age of the patients, and presurgery status were not set as exclusion criteria.

### Data Extraction

All the data were initially screened by two independent authors and another author was required when different opinions existed. The following information from the included studies was extracted: the first author, nationality, year of publication, patients (hips), age, gender, follow-up (years), and outcome parameters. Primary endpoints consist of audible noise, prosthesis fracture, and revision events. Second endpoints include dislocation, deep infection, osteolysis, and prosthesis loosening.

### Quality Assessment

The quality assessment of the trials was performed according to the Cochrane Collaboration's tool and prepared a “risk of bias” table to assess the risk of bias of each included study: random sequence generation, allocation concealment, blinding, incomplete outcome data, selective reporting, and other sources of bias. Three independent authors provided a score of high, unclear, or low risk of bias for each bias domain. When disagreement appeared, the third leading author was involved until the consensus was concluded.

### Statistical Analysis

All the statistical analyses were performed with the Stata 11.0 (StataCorp LP, College Station, Texas, USA). When *I*^2^ < 50% and *p* > 0.1, the data were considered to be with no statistical heterogeneity according to the chi-squared test and *I*^2^ statistic, and a fixed-effects model was adopted for meta-analysis. Otherwise, a random-effects model was performed. The results of dichotomous outcomes (audible noise, prosthesis fracture, revision events, dislocation, infection, osteolysis, and prosthesis loosening) were expressed as odds ratio (OR) with 95% CIs. *p* < 0.05 was considered to be statistically significant.

## Results

### Search Results

**Figure 1:**
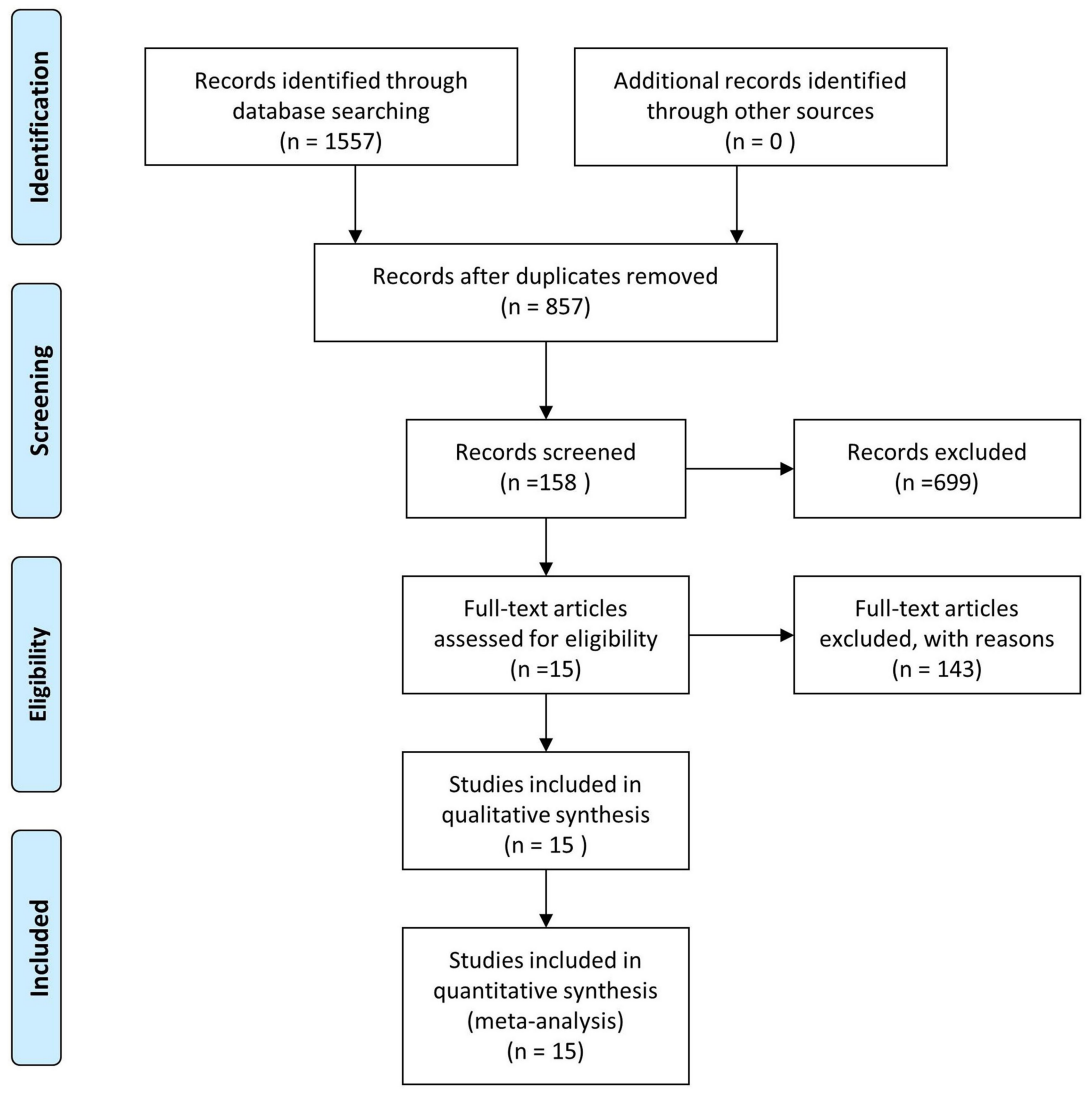
Schematic diagram of the study selection and exclusion procedure for the study.

According to the search strategy, we first identified 1,557 pieces of literature in the 4 selected databases. Subsequently, 700 records were eliminated due to duplication or irrelevant studies. Then, we scanned the title and abstract, 699 reports were excluded based on the eligibility criteria. Thereafter, remaining 158 pieces of literature were reviewed by the full text and 143 reports were further removed for the lack of necessary data. Finally, 15 RCTs were included for the data extraction and meta-analysis (Figure 1).

### Study Characteristics

A total of 15 RCTs published between 2000 and 2020 were included in this study, all of which compared the bearing surfaces of COC and COP in patients receiving THA. A total of 2,169 patients (2,813 hips) were involved, during which 1,481 patients (1,554 hips) participated in the intravenous group and 1,221 patients (1,259 hips) participated in the topical group. Four studies performed THA with these two kinds of bearing surfaces in the same patients (1 hip with COC THA and the other hip with COP THA). Another feature is that the follow-up time in 4 of the studies is more than 10 years. Other demographic characteristics and details such as author, published year, country, enrolment period, mean age, and sex ratio are shown in Table 1. Hip function score was improved significantly in both the groups compared to preoperative values but did not differ significantly between the two groups. Other surgical information is shown in Table 2.

**Table 1:** Characteristics of trials.

**Author Year**	**Country**	**Enrolment**	**Patients (hips)**		**Mean age (y)**		**Gender (M/F)**		**Follow-up (y)**
			**COC**	**COP**	**COC**	**COP**	**COC**	**COP**	
Kim and Park (17)	Korea	1999–2003	133 (133)	133 (133)	53 ± 7	53 ± 7	84/49	84/49	17.1
Atrey et al. (9)	Canada	1997–1999	29 (29)	28 (28)	41.5 ± 8.9	42.8 ± 6.9	17/12	15/13	15
Kim et al. (18)	Korea	2000–2002	100 (100)	100 (100)	45.3	45.3	66/34	66/34	12.4
Beaupre et al. (19)	Canada	1998–2003	48 (48)	44 (44)	51.3 ± 6.9	53.6 ± 6.5	26/22	24/20	10
Cai et al. (20)	China	2008	43 (51)	50 (62)	42.1	42.0	25/18	27/23	3.2
Amanatullah et al. (21)	USA	1999–2001	166 (196)	146 (161)	50.4 ± 12.8	54.7 ± 12.9	106/60	84/62	5
Lewis et al. (22)	Canada	1997–1999	29 (30)	26 (26)	41.5 ± 8.9	42.8 ± 6.9	–	–	8
Lombardi et al. (23)	USA	2000	64 (65)	45 (45)	57	60	35/29	24/21	6
Hamilton et al. (24)	USA	2003	177 (177)	87 (87)	56.4	57.3	90/87	47/40	2.6
Poggie et al. (25)	USA	1999–2003	282 (315)	147 (157)	54	54	–	–	2
Kim et al. (26)	Korea	–	50 (50)	50 (50)	51	51	38/12	38/12	4.8
Bal et al. (27)	USA	1998–2001	250 (250)	250 (250)	54.97 ± 14.7	60.93 ± 12.81	138/112	117/133	2
Nygaard et al. (28)	Denmark	2001–2003	62 (62)	64 (64)	–	–	15/47	26/38	1
Pitto et al. (29)	Germany	–	23 (23)	27 (27)	–	–	–	–	2
Pitto et al. (30)	Germany	–	25 (25)	24 (25)	60 ± 5.5	62 ± 4.5	10/15	8/16	5

*COC, Ceramic on Ceramic; COP, Ceramic on Polyethylene; M, male; F, female*.

**Table 2:** Characteristics of included trials showing general surgical information.

**Author Year**	**Surgical approach**	**Prosthesis (femoral head-acetabular liner)**		**Head diameter (mm)**	**Hip function score**			
					**COC**		**COP**	
		**Ceramic-Ceramic (COC)**	**Ceramic-Polyethylene (COP)**		**Pre-OP**	**Current**	**Pre-OP**	**Current**
Kim and Park (17)	Posterolateral	Alumina-Alumina Ceramic	Alumina Ceramic- HXLPE	28	HHS: 39 ± 11	HHS: 94 ± 5	HHS: 41 ± 10	HHS: 91.6 ± 5
Atrey et al. (9)	–	Alumina-Alumina Ceramic	Alumina Ceramic-UHMWPE	28	HHS: 50.3 ± 13.7	HHS: 94.2 ± 6.9	HHS: 48.8 ± 19.9	HHS: 91.6 ± 7.2
Kim et al. (18)	Posterolateral	Alumina-Alumina Ceramic	Alumina Ceramic-HCL PE	28	HHS:38	HHS:94	HHS:37	HHS:95
Beaupre et al. (19)	Hardinge or Posterolateral	Alumina-Alumina Ceramic	Alumina Ceramic-HCL PE	28 or 32	WOMAC: 46.0 ± 11.1	WOMAC: 82.5 ± 18.3	WOMAC:47.0 ± 19.4	WOMAC: 86.6 ± 17.1
Cai et al. (20)	Posterolateral	Delta-Delta Ceramic	Alumina Ceramic-UCL PE	28 or 36	HHS improvement: 36.4 ± 8.0		HHS improvement: 37.0 ± 8.2	
Amanatullah et al. (21)	–	Alumina-Alumina Ceramic	Alumina Ceramic-UCL PE	28 or 32	HHS: 43 ± 10	HHS: 91 ± 27	HHS: 43 ± 10	HHS: 91 ± 27
Lewis et al. (22)	Posterior	Alumina-Alumina Ceramic	Alumina Ceramic-UCL PE	28	SMH:15.8	SMH:21.1	SMH:16.2	SMH:19.5
Lombardi et al. (23)	Lateral	Delta-Alumina Ceramic	Zirconia Ceramic-HCL PE	28 or 32	HHS:51	HHS:90	HHS:48	HHS:92
Hamilton et al. (24)	Lateral or Posterior	Delta-Delta Ceramic	Delta Ceramic-MCL PE	28	HHS:50.6	HHS:94.4	HHS:50.7	HHS:93.8
Poggie et al. (25)	–	Alumina-Alumina Ceramic	Alumina Ceramic-UCL PE	28	HHS:45	HHS:92	HHS:43	HHS:93
Kim et al. (26)	–	Alumina-Alumina Ceramic	Alumina Ceramic-UCL PE	28	HHS:46	HHS:93	HHS:46	HHS:93
Bal et al. (27)	–	Alumina-Alumina Ceramic	Alumina Ceramic-PE (UC)	28	–	–	–	–
Nygaard et al. (28)	Postlateral	Alumina-Alumina Ceramic	Zirconia Ceramic-UCL PE	28	–	–	–	–
Pitto et al. (29)	–	Alumina-Alumina Ceramic	Alumina Ceramic-UCL PE	28	HHS:48.9	HHS:94.1	HHS:47.7	HHS:93.7
Pitto et al. (30)	–	Alumina-Alumina Ceramic	Alumina Ceramic-PE (UC)	28	HHS:53	HHS:94.5	HHS:53	HHS:94.5

*PE, Polyethylene; HXLPE, highly cross-linked polyethylene; UHMWPE, ultra-high molecular weight polyethylene; HCL, highly cross-linked; MCL, moderately cross-linked; UCL, uncross-linked ultra-high-molecular-weight; SMH, St. Michael's Hospital scores; HSS, harris hip score; WOMAC, Western Ontario McMaster Osteoarthritis Index; UC, unclear*.

### Risk of Bias

The risk of bias of the included RCTs was assessed by two independent authors. Random sequence generation was illustrated in 8 studies. Allocation concealment and blinding of participants and personnel were considered with a low risk of bias in 6 studies. All 15 studies were thought to have a low risk of bias on the blinding of outcome assessment. Besides, all the 15 RCTs did not make selective reports (Table 3).

**Table 3:** Cochrane Collaboration's tool for quality assessment in trials comparing COC to COP.

**Trials**	**Sequence generation**	**Allocation concealment**	**Blinding of outcome assessors**	**Incomplete outcome data**	**Selective outcome reporting**	**Others**
Kim and Park (17)	Low	low	Low	Low	Low	Low
Atrey et al. (9)	Unclear	Unclear	Low	Low	Low	Unclear
Kim et al. (18)	Low	Unclear	Low	Low	Low	Low
Beaupre et al. (19)	Low	Low	Low	Low	Low	Low
Cai et al. (20)	Unclear	Unclear	Low	Low	Low	Low
Amanatullah et al. (21)	Low	Low	Low	Low	Low	Low
Lewis et al. (22)	Unclear	Unclear	Low	Low	Low	Low
Lombardi et al. (23)	Low	Low	Low	Low	Low	Unclear
Hamilton et al. (24)	Low	Unclear	Low	Low	Low	Low
Poggie et al. (25)	Unclear	Unclear	Low	Low	Low	Low
Kim et al. (26)	Low	Low	Low	High	Low	Unclear
Bal et al. (27)	Unclear	Unclear	Low	Low	Low	High
Nygaard et al. (28)	Low	Low	Low	Low	Low	Low
Pitto et al. (29)	Unclear	Unclear	Low	High	Low	High
Pitto et al. (30)	Unclear	Unclear	Low	High	Low	High

*COC, Ceramic-Ceramic; COP, Ceramic-Polyethylene*.

### Outcomes of the Meta-Analysis

The outcomes of COC vs. COP during THA were assessed *via* the evaluation indicators including audible noise, prosthesis fracture, and revision events, dislocation, deep infection, osteolysis, and prosthesis loosening.

### Audible Noise

**Figure 2:**
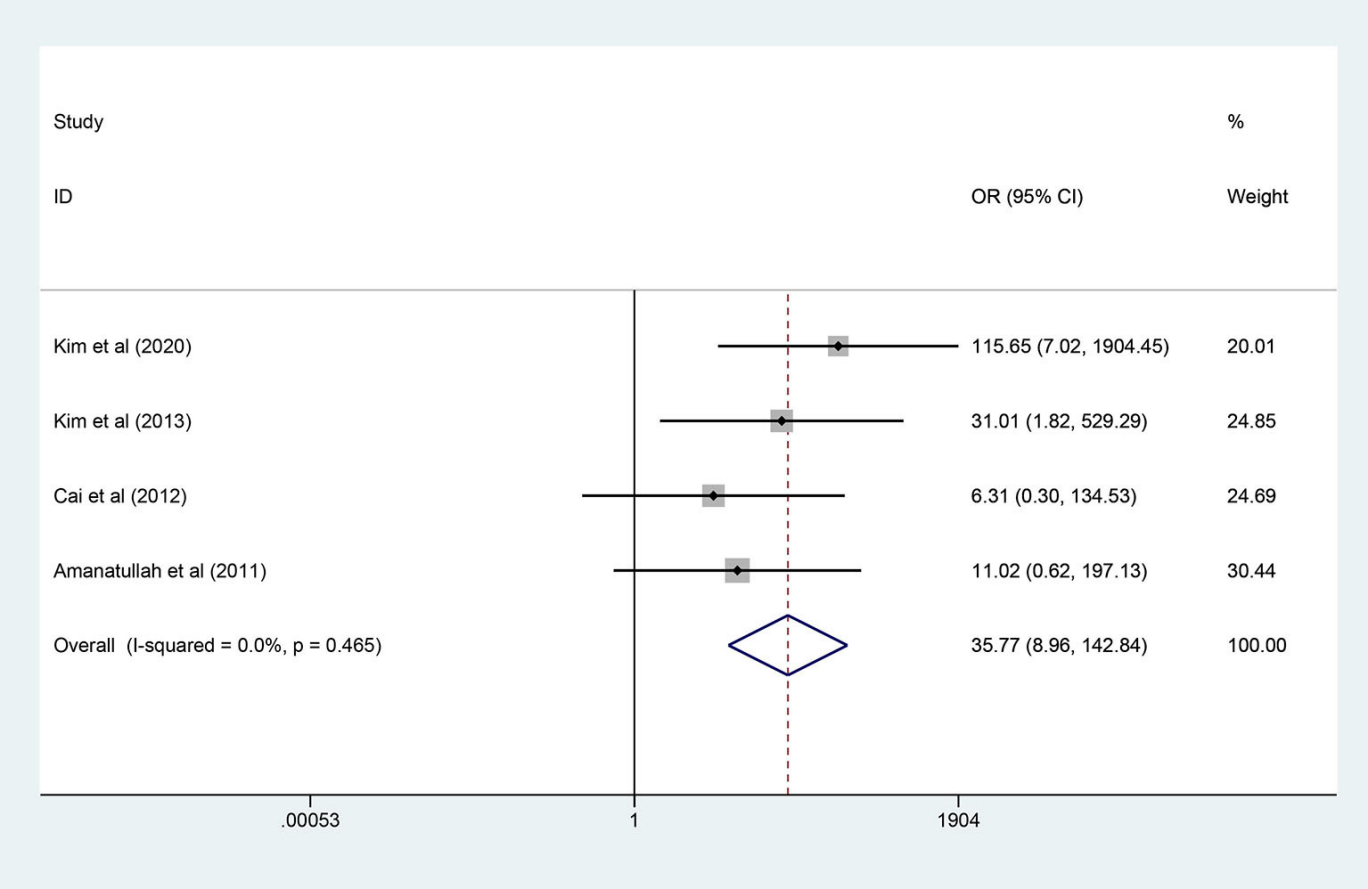
Forest plot diagram of the audible noise rate of the replaced hip with ceramic-on-ceramic (COC) and ceramic-on-polyethylene (COP) bearing surfaces.

A total of 4 RCTs reported the data of audible noise (described as squeaking or clicking sound) after THA, during which 61 of 480 hips in the COC group were positive and no cases in the COP group. The data from different studies did not reveal significant heterogeneity (*p* = 0.465, *I*^2^ = 0.0%). As shown in Figure 2, the component-related noise occurred more frequently in COC than COP and the difference was statistically significant (OR = 5.919; 95% CI: 2.043, 17.146; *p* ≤ 0.001).

### Prosthesis Fracture

**Figure 3:**
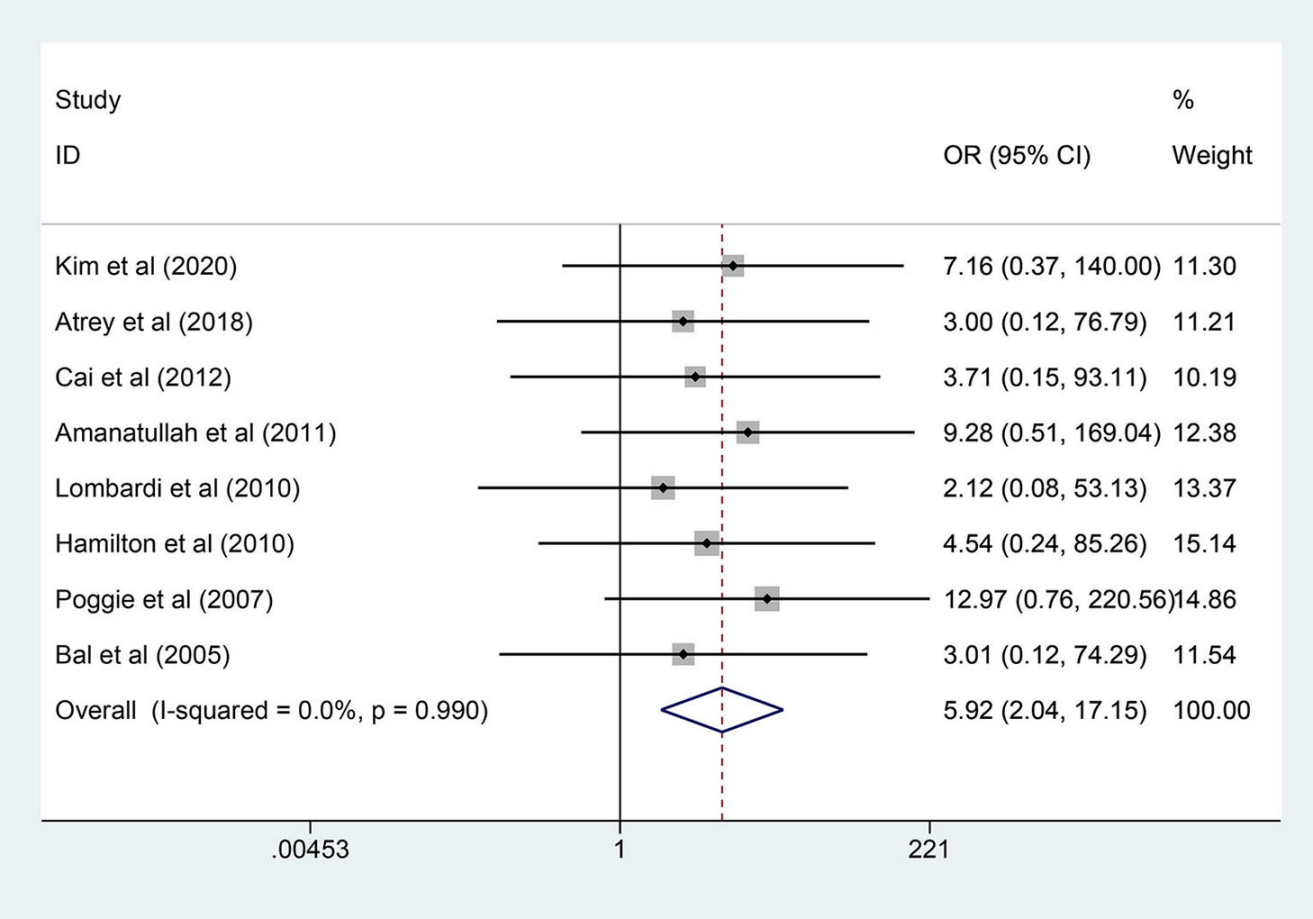
Forest plot diagram of the prosthesis fracture rate of the replaced hip with COC and COP bearing surfaces.

A total of 8 RCTs reported the events of prosthesis fracture, during which 28 of 1,188 hips in the COC group were positive and no cases in the COP group. The data from different studies did not reveal significant heterogeneity (*p* = 0.990, *I*^2^ = 0.0%). As shown in Figure 3, the prosthesis fracture occurred more frequently in COC than COP and the difference was statistically significant (OR = 35.768; 95% CI: 8.957, 142.836; *p* = 0.001).

### Revision

**Figure 4:**
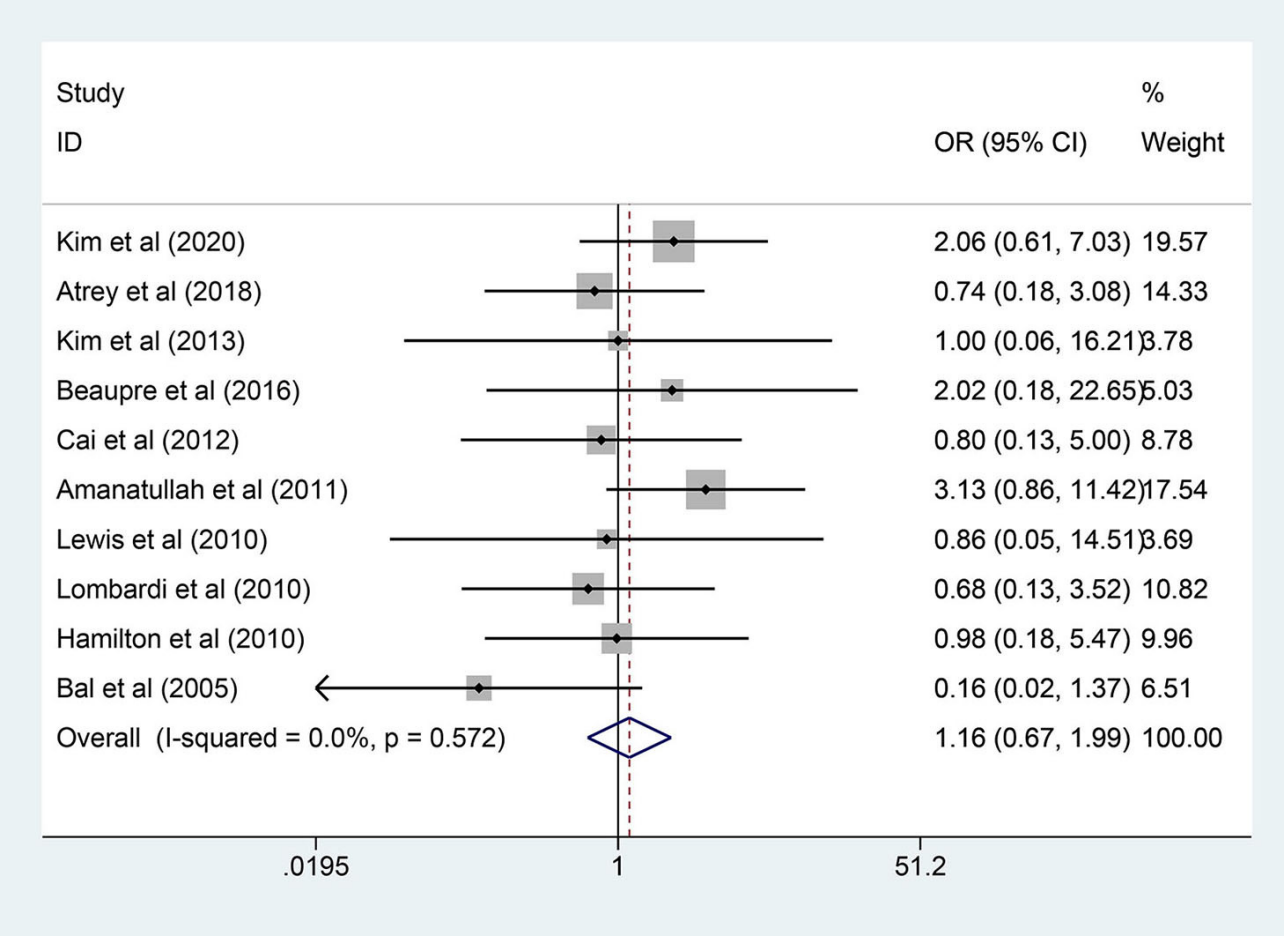
Forest plot diagram of the overall revision rate of the replaced hip with COC and COP bearing surfaces.

A total of 10 RCTs reported direct information on the revision events. The revision was needed in 37 of 1,094 hips (3.4%) in the COC group compared to 29 of 963 hips (3.0%) in the COP group. Significant heterogeneity was not revealed (*I*^2^ = 0.0%, *p* = 0.572), so a fixed-effects model was adopted. As shown in Figure 4, the results showed that there was no significant difference in the overall revision rate between the two groups (OR = 1.158; 95% CI: 0.674, 1.991; *p* = 0.595).

A subgroup analysis was further performed based on follow-up years (≥10 years) or follow-up years (<10 years). Among the whole 10 RCTs, the follow-up time of 4 studies (347 patients) was longer than 10 years, while the follow-up time of 6 studies (747 patients) was <10 years. A fixed-effects model was adopted because significant heterogeneity was not found among the studies (*I*^2^ < 50%, *p* > 0.1). Similar to the overall analysis, neither of these two subgroups showed a significant difference in the revision rate. More details are shown in Table 4.

**Table 4:** The postoperative outcomes of this meta-analysis.

**Outcomes**	**Studies numbers**	**Groups size**		**Overall effect**			**Heterogeneity**	
		**COC**	**COP**	**Effect estimate**	**95% CI**	***P*-value**	***I*^2^(%)**	***P*-value**
Audible noise	4	419	456	OR, 35.768	8.957, 142.836	0.000	0.0	0.465
Prosthesis fracture	8	1,188	923	OR, 5.919	2.043, 17.146	0.001	0.0	0.990
Revision	10	1,094	963	OR, 1.158	0.674, 1.991	0.595	0.0	0.572
≥ 10 y	4	347	350	OR, 1.366	0.596, 3.130	0.461	0.0	0.729
<10 y	6	747	613	OR, 1.024	0.500, 2.095	0.948	17.5	0.300
Dislocation	11	1,360	1,057	OR, 0.748	0.457, 1.223	0.247	0.0	0.990
Deep infection	8	938	805	RD, 0.001	−0.005, 0.008	0.720	0.0	0.881
≥ 10 y	3	204	203	RD, 0.007	−0.018, 0.032	0.585	0.0	0.797
<10 y	5	734	602	RD, 0.001	−0.006, 0.008	0.824	0.0	0.669
Osteolysis	11	1,390	1,083	RD, −0.001	−0.006, 0.004	0.771	0.0	0.732
≥ 10 y	4	308	301	RD, −0.001	−0.012, 0.010	0.872	25.0	0.261
<10 y	7	1,082	782	RD, −0.001	−0.006, 0.005	0.806	0.0	0.817
Prosthesis loosening	9	1,037	901	RD, −0.000	−0.007, 0.007	0.962	0.0	0.855

*COC, Ceramic-Ceramic; COP, Ceramic-Polyethylene; CI, confidence interval; RR, risk ratio; WMD, weighted mean difference*.

### Dislocation

**Figure 5:**
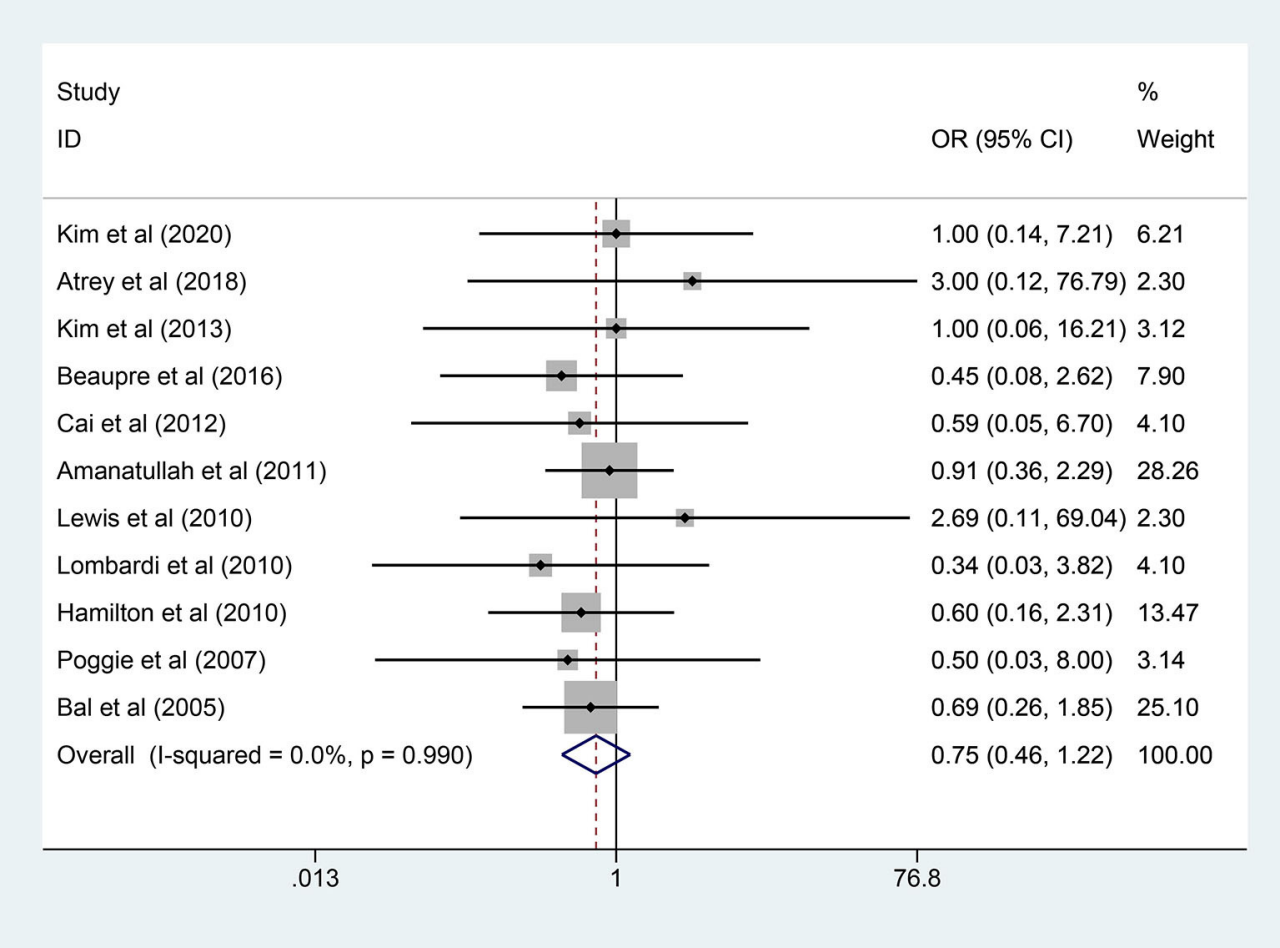
Forest plot diagram of the dislocation rate of the replaced hip with COC and COP bearing surfaces.

A total of 11 studies (2,417 hips) mentioned the data of dislocation and a fixed-effects model was used owing to no significant heterogeneity among the studies (*I*^2^ = 0.0%, *p* = 0.990). As shown in Figure 5, the results demonstrated that there was no significant difference in dislocation rate between the two groups (OR = 0.748; 95% CI: 0.457, 1.223; *p* = 0.247).

### Deep Infection

**Figure 6:**
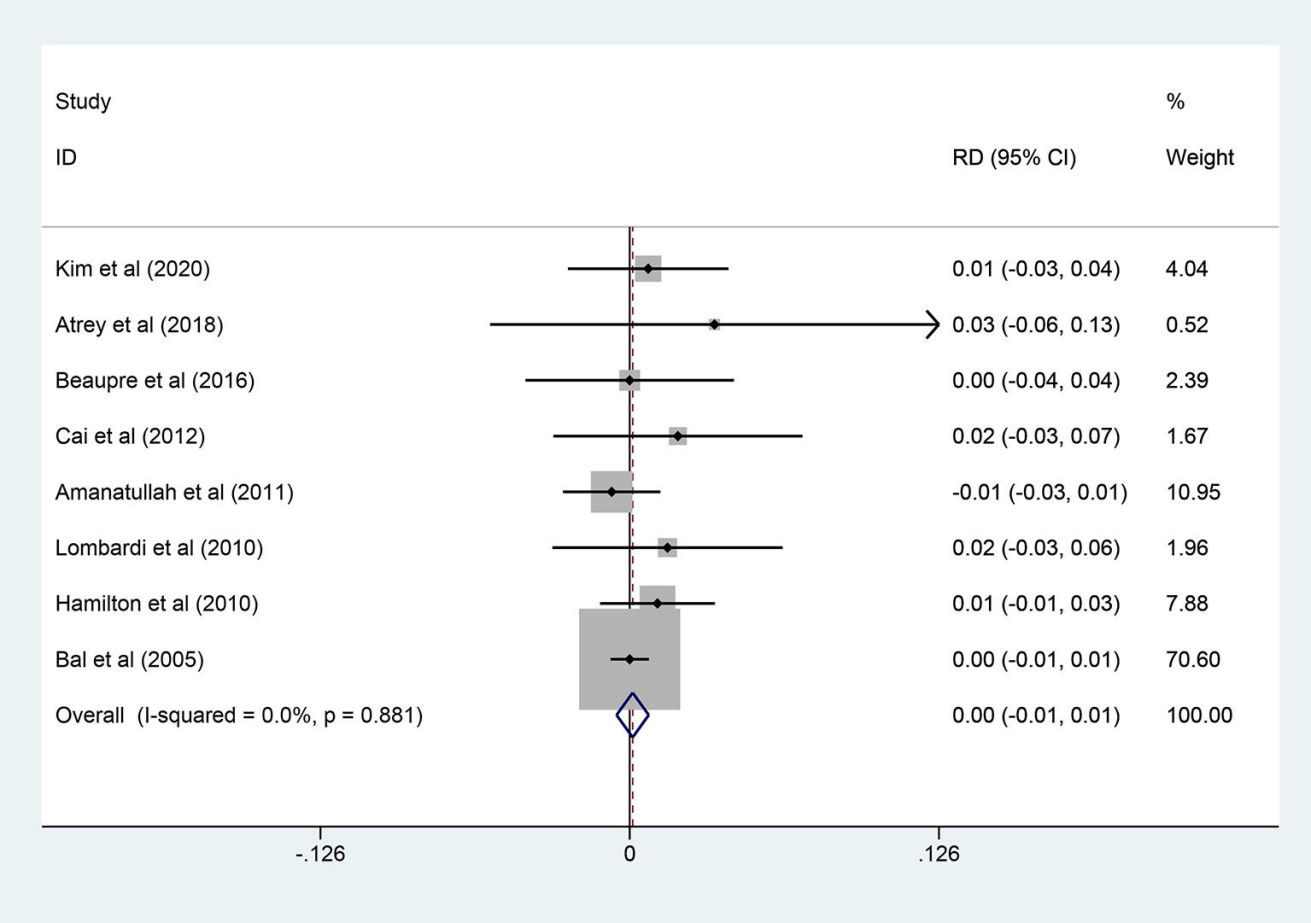
Forest plot diagram of the overall deep infection rate of the replaced hip with COC and COP bearing surfaces.

A total of 8 publications provided the numbers of deep infection events of the patient after THA and there was no significant heterogeneity (*I*^2^ = 0.0%, *p* = 0.881). Compared to 938 hips in the COC group to 805 hips in the COP group, there was no statistical difference in overall deep infection rate between the two groups (RD = 0.001; 95% CI: −0.005, 0.008; *p* = 0.720, Figure 6). As for the subgroup analysis, there was also no statistical difference in either the 3 studies with follow-up years (≥10 years) or the 5 studies with follow-up years (<10 years). More details are shown in Table 4.

### Osteolysis

**Figure 7:**
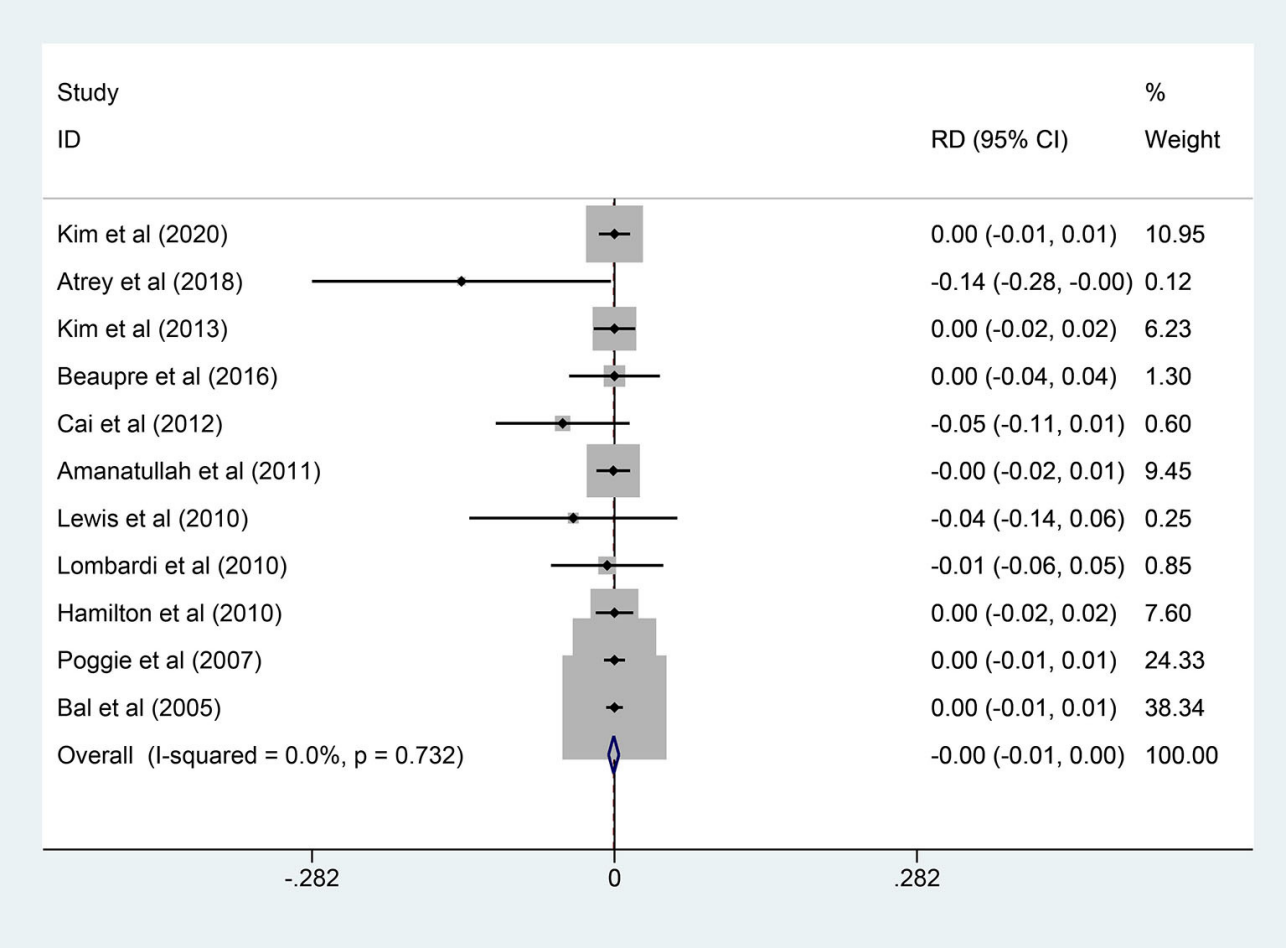
Forest plot diagram of the overall osteolysis rate of the replaced hip with COC and COP bearing surfaces.

A total of 11 studies (928 patients) reported osteolysis cases. A fixed-effects model was adopted due to the non-significant heterogeneity (*I*^2^ = 0.0%, *p* = 0.732). As shown in Figure 7, pooled results revealed no statistical difference in the two groups (RD = −0.001; 95% CI: −0.006, 0.004; *p* = 0.771). Similarly, there was no statistical difference in the subgroup analysis. More details are shown in Table 4.

### Prosthesis Loosening

**Figure 8:**
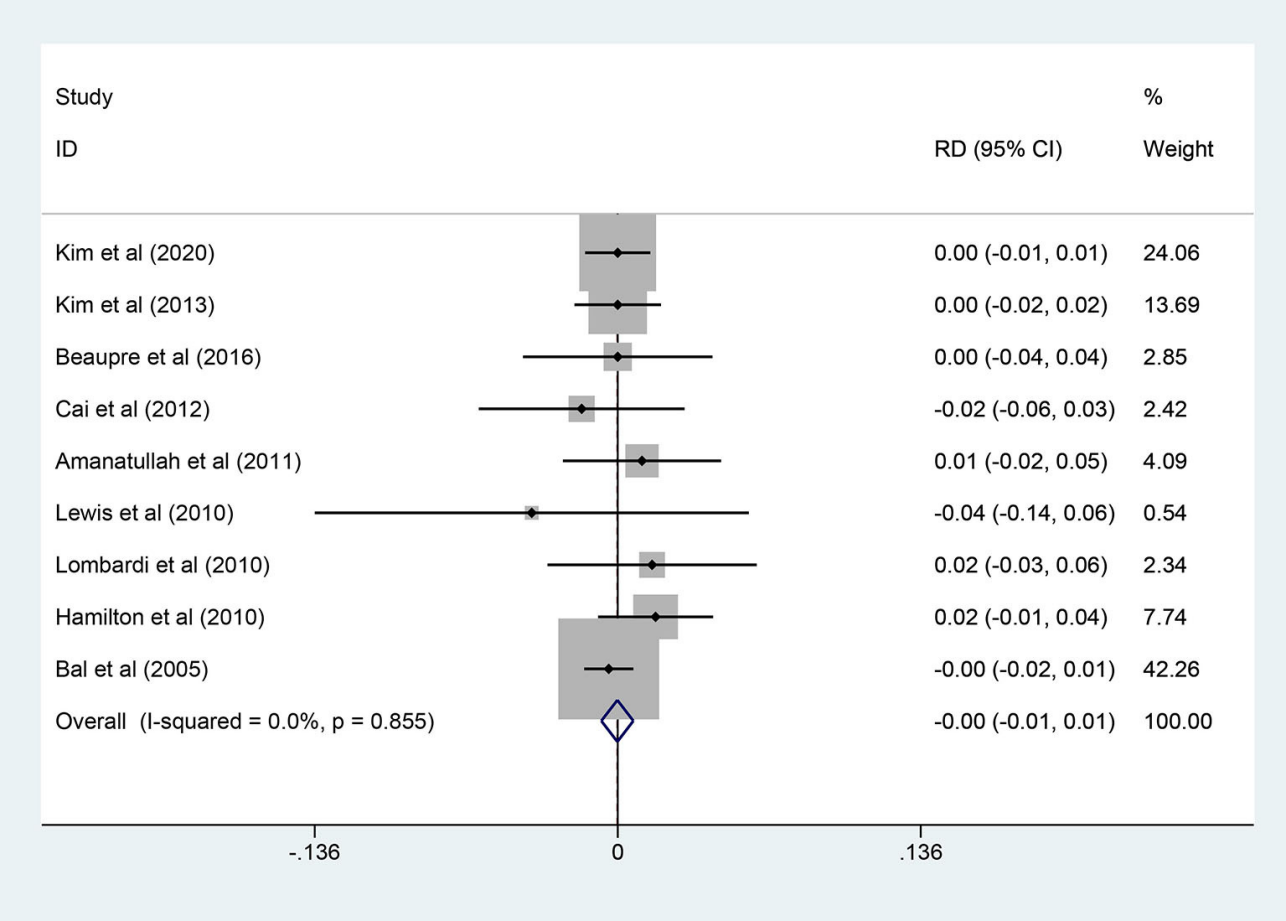
Forest plot diagram of the prosthesis loosening rate of the replaced hip with COC and COP bearing surfaces.

A total of 9 studies (1,037 hips) reported prosthesis loosening events and a fixed-effects model was used owing to non-significant heterogeneity among the studies (*I*^2^ = 0.0%, *p* = 0.855). As shown in Figure 8, the results demonstrated that there was no significant difference in prosthesis loosening rate between the two groups (RD = −0.000; 95% CI: −0.007, 0.007; *p* = 0.962).

## Discussion

Although many factors such as surgical technique, the status of the patient, and positioning of the prosthesis could affect the success of the surgery and the long-term survival of the artificial joint, prosthetic material, especially the selection of optimal bearing surface for the patient, must be considered by a surgeon due to different advantages and drawbacks of various types of bearing surfaces. Currently, the most commonly used bearing surfaces are COC and COP in clinical, both of which have shown reliable clinical results. COC bearing surface is attractive mainly owing to the low friction and low wear rates compared with the COP. Meanwhile, COC has material-related shortcomings such as the risk of fracture and the occurrence of audible noises. Despite previous clinical trials and meta-analyses having compared and evaluated these two kinds of bearing surfaces, the long-term follow-up data (≥10 years) are lacking. Therefore, we performed the meta-analysis to compare the COC with the COP according to included 15 RCTs and further subgroup analysis of the long-term follow-up studies.

The most important issue in the modern THA is the revision rate, which is mainly caused by wear debris-induced osteolysis, implant loosening, or deep infection. In this study, there were no significant differences between the two groups and the same result also appeared in the long-term follow-up studies. However, it is known that COC is with lower wear rate compared with COP due to the hardness and high wettability of the character of the material itself (31, 32). During the included RCTs, 5 studies reported the wear rate of bearing surface and all of them revealed that the mean liner wear rate in the COC group was lower than that in the COP group (9, 17, 18, 21, 22). In theory, a higher wear rate means more wear debris, which could induce osteolysis, implant loosening, or deep infection. Intriguingly, all of the three revision-related events were demonstrated, as no statistical difference in the two groups. Ceramic wear debris has shown to be less biologically active in laboratory studies (33). Therefore, the explanation that ceramic wear particles may be fewer in number, but more reactive than PE cannot be convincing. Considering the metal debris from the taper-trunnion junction in hip prostheses has been widely recognized (34), it is reasonable to speculate the COC with a lower wear rate can produce a considerable number of metal debris from the taper-trunnion junction, which may compromise the advantage of COC on wear rate. More importantly, except for the bearing surfaces, other reasons such as the diameter of the femoral head (4), the position of the acetabular cup (35), and the prosthesis from different manufacturers (36) can also affect the final result.

The COC bearing has two main drawbacks that hinder its widespread use. First, as a catastrophic complication, the brittleness of ceramics theoretically increases the risk of prosthesis fracture. Interestingly, ceramics fracture was mostly reported to appear in the liner with percentages between 0.13 and 1.1% and only occasionally occur in the head (37). In this study, COC bearing demonstrated a significantly higher prosthesis fracture rate compared with COP bearing and all the prosthesis fracture events were reported to happen in the liner (9, 17, 20, 21, 23–25, 27). Although it is easy to understand the fracture of the ceramic caused by exogenous violence, the liner fracture is rarely related to direct trauma. Instead, misalignment during insertion of the liner, metal back damage, or acetabular component malposition are the common reasons that lead to liner fracture (38, 39). Thus, it is reasonable to be careful in the preparation of the acetabulum and insertion of the liner when choosing the ceramic liner, while the PE liner seems to have a relatively higher “fault tolerance.” The second drawback of COC bearing is the audible noises (squeaking or clicking) and it is often complained about by patients after THA. In this meta-analysis, 4 studies reported the noise events and the positive rate was 14.6% in the COC group, while no hips were reported in the COP group. Most of the noises were caused by the separation of the head from the liner due to postoperative soft-tissue laxity and will subside automatically in some days, while only very few cases were caused by the real ceramic friction. More importantly, the surgeon needs to keep in mind that the occurrence of a delayed noise in a COC joint, especially accompanied by pain and malposition, can be caused by the fracture of ceramics (40).

Another issue after THA was the dislocation. Although the dislocation rate was not significantly different in the two groups, confounding factors could have interfered with the results. Both the femoral head size and liner design could affect the dislocation rate, except for the position of the acetabular cup. “Protective liners” including lipped liners (41) and constrained liners (42) could significantly reduce the risk of dislocation. However, to some extent, constrained liners will affect the range of joint motion and it is easier for impingement to happen compared with normal liners (43). More importantly, constrained liners should not be used as a remedial measure for defects in surgical technique or prosthesis placement. Although COC bearing was limited in the choice of the constrained liner because of the material properties, it was allowed to choose a larger head size. It was reported that a larger head could remarkably reduce dislocation by increasing impingement-free range of hip movement and jumping distance (44). However, larger diameter heads were theoretically associated with increased wear and revision rate, although long-term follow-up studies about this term were rare.

Despite the present meta-analysis including the most comprehensive RCTs and 4 of them reported long-term (≥10 years) follow-up results after THA, there are still some limitations. First of all, prostheses used in different trials were produced by different manufacturers and were of different sizes, all of which may result in bias. Second, outcomes parameters such as wear rate and hip function were different in different studies and cannot be pooled for further analysis. In addition, high-quality RCTs with a long-term follow-up are still urgently needed to compare the effect of these two bearing surfaces.

## Conclusion

This meta-analysis indicated the comparable functional outcomes of COC and COP bearing surfaces following primary THA. There were statistically significant differences in component-related noise and ceramic component fracture between the two groups. Both the overall and subtype analysis showed similar dislocation rate, deep infection rate, osteolysis rate, prosthesis loosening rate, and revision rate. Considering the amount of included RCTs is limited, the generality of our conclusion is relatively restricted. Therefore, more high-quality RCTs are necessary to support the existing conclusion in the future.

## Strengths and Limitations of This Study

Strengths: (1) The present meta-analysis included the most comprehensive randomized controlled trials (RCTs) on the “head-to-head” comparison of ceramic-on-ceramic (COC) components and ceramic-on-polyethylene (COP) components during total hip arthroplasty (THA); (2) Four of the trials reported long-term (≥10 years) follow-up results after THA, which increased the reliability of the results; and (3) This study could provide strong evidence for clinicians to make decisions on the choice of bearing surfaces during THA.

Limitations: (1) Prostheses used in different trials were produced by different manufacturers and were of different sizes, all of which may result in bias; (2) Outcome parameters such as wear rate and hip function were different in studies and cannot be pooled for further analysis; (3) High-quality RCTs with a long-term follow-up are still urgently needed to compare the effect of these two bearing surfaces.

## Data Availability Statement

The original contributions presented in the study are included in the article/supplementary material, further inquiries can be directed to the corresponding author/s.

## Author Contributions

YF and XS: conceptualization, investigation, resources, and visualization. YF: writing—original draft preparation. XS: writing—review and editing. All authors have read and agreed to the published version of the manuscript.

## Conflict of Interest

The authors declare that the research was conducted in the absence of any commercial or financial relationships that could be construed as a potential conflict of interest.

## Publisher's Note

All claims expressed in this article are solely those of the authors and do not necessarily represent those of their affiliated organizations, or those of the publisher, the editors and the reviewers. Any product that may be evaluated in this article, or claim that may be made by its manufacturer, is not guaranteed or endorsed by the publisher.
