# Corrigendum: Comparison of Ceramic-on-Ceramic vs. Ceramic-on-Polyethylene for Primary Total Hip Arthroplasty: A Meta-Analysis of 15 Randomized Trials

**DOI:** 10.3389/fsurg.2022.876080

**Published:** 2022-03-14

**Authors:** Yan Fang, Xiaobin Shang

**Affiliations:** ^1^Department of Anesthesiology, Tongji Hospital, Tongji Medical College, Huazhong University of Science and Technology, Wuhan, China; ^2^Department of Orthopaedics, Tongji Hospital, Tongji Medical College, Huazhong University of Science and Technology, Wuhan, China

**Keywords:** ceramic on ceramic, ceramic on polyethylene, meta-analysis, total hip, review–systematic

For personal reasons, we have decided to change the corresponding author. The updated corresponding author would be ^**^Xiaobin Shang, xiaobinshang0929@gmail.com^**^.

The authors apologize for this thing and state that Xiaobin Shang did a great contribution to the article and is qualified to be the corresponding author. The original article has been updated.

## Publisher's Note

All claims expressed in this article are solely those of the authors and do not necessarily represent those of their affiliated organizations, or those of the publisher, the editors and the reviewers. Any product that may be evaluated in this article, or claim that may be made by its manufacturer, is not guaranteed or endorsed by the publisher.

